# Doing Much

**Published:** 1874-09

**Authors:** 


### DOING MUCH

Many persons seem to be always in a hurry, and yet never accomplish much; others never to be hurried, and yet do a very great deal. If you have fifty letters to answer, don't waste time in looking over to find which one should be noticed first; answer the one you first lay your hands on, and then go through the whole pile. Some begin a thing and leave it partially completed, and hurry off to something else. A better plan is to complete whatever you undertake before you leave it, and be thorough in everything; it is the going back from one thing to another that wastes valuable time. Another thing, deliberate workers are those who accomplish the most work in a given time and are less tired at the end of the day than many who have not accomplished half as much; the hurried worker has often to do his work twice over, and even then it is seldom done in the best manner, either as to neatness or durability. It is the deliberate and measured expenditure of strength which invigorates the constitution and builds up the health; multitudes of firemen have found an early death, while the plough-boy lives healthily and lives long, going down to his grave beyond three-score and ten.

